# Association between Ag-RP, alpha-MSH and cardiovascular risk factors regarding adherence to diet quality index-international (DQI-I) among obese individuals

**DOI:** 10.34172/jcvtr.2021.48

**Published:** 2021-11-28

**Authors:** Mahsa Mahmoudinezhad, Mahdieh Abbasalizad Farhangi

**Affiliations:** Drug Applied Research Center, Department of Community Nutrition, Faculty of Nutrition, Tabriz University of Medical Sciences, Tabriz, Iran

**Keywords:** Ag-RP, Alpha-MSH, DQI-I, Obesity, Overweight

## Abstract

***Introduction:*** Obesity is a strong promoter of cardiometabolic risk factors and is associated with several chronic comorbidities. Recently, the role of α-melanocyte stimulating hormone (α-MSH) and agouti related peptide (Ag-RP) in regulation of energy balance has attracted much attention. In current study, we evaluated the association between α-MSH and Ag-RP with cardiometabolic factors among obese individuals with different adherence to Diet Quality Index-International (DQI-I) values.

***Methods:*** In this research, 188 obese adults aged between 20 and 50 years old and body mass index (BMI) between 30 and 40 kg/m^2^ were recruited. Dietary intakes of participants and DQI-I calculation was performed using a semi-quantitative food frequency questionnaire (FFQ) with 132 food items. Serum glucose, lipids, insulin, and plasma α-MSH and Ag-RP levels were measured using ELISA kits. Homeostasis model assessment for insulin resistance index (HOMA-IR) and quantitative insulin sensitivity check index (QUICKI) were also calculated.

***Results:*** Among those with the lowest adherence to DQI-I, Ag-RP was positively associated with systolic blood pressure (SBP) (*P* = 0.03) among males, which was associated with waist circumference (WC) (*P* = 0.01) and diastolic blood pressure (DBP) (*P* = 0.01). Moreover, among males with low and moderate adherence to DQI-I, α-MSH was positively associated with insulin (*P* = 0.04), weight (*P* = 0.03), WC (*P* < 0.01), SDP (*P* = 0.02) and DBP (*P* = 0.01). Also, Ag-RP showed a positive association with BMI values (R^2^ = 0.03; *P* = 0.03).

***Conclusion:*** According to our findings, in obese subjects with poor to moderate adherence to DQI-I, Ag-RP and α-MSH were in positive correlation with cardiometabolic risk factors. These findings further clarify the clinical importance of these parameters as prognostic factors of cardiometabolic abnormalities.

## Introduction

Obesity is a global public health challenge and its trend in Iran has reached an alarming point, so that epidemiological studies have reported 22.3% prevalence of obesity among Iranian population.^1^ Despite growing production of scientific knowledge about the etiology and pathophysiology of obesity, its prevalence is still high. Obesity is a strong promoter of cardiometabolic risk factors and it is associated with growing prevalence of chronic diseases, including cardiovascular events, metabolic syndrome, diabetes, sleep disorders, and cancer; therefore, it is important to address the metabolic mediators involved in pathophysiology of obesity.^2,3^ Previous studies have demonstrated that obesity results from multiple interactions between several anorexigenic and orexigenic neuropeptides released by hypothalamic arcuate nucleus (ARC) neurons and are involved in energy hemostasis and feeding control.^4,5^ Anorexigenic and orexigenic neurons exert their obesity-accelerating effect via proopiomelanocortin (POMC) and agouti-related peptide (Ag-RP) gene expression.^6,7^ Also, α-melanocyte-stimulating hormone (α-MSH), generated from POMC, sends numerous signals to reduce energy intake.^8^ Following food intake, leptin reduces food intake and increases energy consumption via stimulating α-MSH and inhibiting Ag-RP production.^9^ Similarly, Ag-RP also exhibits its effect in reducing energy expenditure and increasing appetite.^7,10^ Moreover, Ag-RP neuropeptide antagonizes α-MSH effects and leads to lipogenesis and reduced energy consumption.^11^ It has been reported that disturbance in the above mentioned pathways is responsible in obesity promotion. Several experimental studies have indicated that impaired melanocortin system is associated with obesity, hyperglycemia, and hyperinsulinemia.^12^ In addition, previous studies have reported the effect of these two neuropeptides on regulation of body weight, serum leptin level, and adiposity level.^8,10^ Katsuki et al reported that α-MSH significantly increased among obese males compared to non-obese ones; also, α-MSH level was positively correlated with BMI, fasting insulin levels, and visceral fat area.^13^ In a study by Wirth MM et al, injecting α-MSH to brain of mice reduced neuropeptide Y (NPY) effects on increasing food intake.^14^ Similarly, studies have shown that α-MSH injection in obese POMC knockout rats led to weight loss.^15^ Wirth *et al* reported that increased Ag-RP expression leads to severe obesity and hyperglycemia.^14^ In addition, Ag-RP may decrease uncoupling protein-1 (UCP-1) expression and thermogenesis in brown adipose tissue and inhibit sympathetic activity in mice.^16,17^ Several human studies have also revealed increased plasma levels of these two hormones among obese individuals.^18,19^ On the other hand, lifestyle modification, such as change in dietary behavior, has an important role in the prevention and control of obesity and cardiometabolic risk factors.^20^ While previous studies in this context have focused on isolate actions of dietary nutrients, in a usual diet, numerous nutrients are eaten altogether. Therefore, the interactive and synergistic effects of dietary nutrients have motivated researchers to evaluate the approach towards dietary patterns and indices. The American Dietetic Association (ADA) suggests that nutritional messages for general public should consider the whole diet or dietary pattern, rather than emphasizing on consuming a single nutrient.^21^ This combined approach can be used as an alternative or complementary method in nutritional epidemiology to extract the relationship between diet and cardiometabolic risk factors. Recently, several dietary approaches like diet quality index-international (DQI-I) have been developed to investigate dietary variations and their association with metabolic factors.^22,23^ Therefore, in current study, we evaluated the association between α-MSH and Ag-RP with cardiometabolic risk factors among apparently healthy obese individuals with different levels of adherence to DQI-I.

## Materials and Methods

### Study design and participants

In this cross-sectional study, we investigated the relationship between Ag-RP, α-MSH, and cardiometabolic risk factors in Tabriz, Iran from 2017 to 2019. Using simple random sampling method, a total of 188 apparently healthy obese individuals aged 20-50 years old (body mass index (BMI) range: 30-40 kg/m^2^)were included. Individuals with chronic diseases, such as diabetes mellitus, cancers, cardiovascular and renal diseases, and thyroid disorders were excluded from the study. We also excluded pregnant and lactating women, subjects who had followed a dietary program and experienced a significant change in body weight in the last six month, or those who had consumed drugs with significant effect on body weight such as corticosteroids. The sample size calculation was based on the association of obesity with Ag-RP and α-MSH. For this purpose, using G-power software, considering correlation coefficient (r) of 0.25, α = 0.05 and β = 0.20, and power 80%, the minimum sample size was estimated to be 160. Finally, considering 15% drop-out, the final sample size was 188 individuals. According to the estimated power and r values, we decided to subgroup the individuals into tertiles for maintaining the study’s power of 80% and for assigning the comparable number of participants to each group (e.g., at least 55 individuals in each group according to the estimated power of 80%). All participants signed a written informed consent prior to participation and they were free to leave the study at any stage.

### Demographic and anthropometric assessments

Demographic information, including age, medical history, and drug use were obtained using a structured questionnaire. Socioeconomic status (SES), including information about educational attainment, occupation, marital status, and family size were obtained from participants and the total SES score was calculated. Body weight, height, BMI, and waist circumference (WC) were assessed. Body weight was measured using Seca scale to the nearest 100 gr while subjects removed their shoes and wore lightweight cloths. Height was measured on a standing position with distributing weight and four points of body including head, shoulder, buttocks, and heels on the stadiometer’s vertical backboard to the nearest 0.5 cm.^24^ WC was measured using non-stretchable tape at the narrow part of abdomen.

### Physical activity assessment

Short form of International Physical Activity Questionnaire (IPAQ) was used to evaluate the physical activity level of participants.^25^

### Blood pressure

Systolic blood pressure (SBP) and diastolic blood pressure (DBP) were assessed in a relaxing and sitting position using sphygmomanometer after resting for 15 minutes. BP measurement was done twice and the mean of two measurements were reported.

### Appetite assessments

Appetite was measured using the 100-mm Visual Analog Scale (VAS) questionnaire, which includes questions about feeling of hunger, fullness, satiation, and desire to eat sweet, salty, or fatty foods.^26^ All participants were asked to make a mark on 100-mm line based on their feeling. Likewise, the distance from the left side of the line up to the mark determined the final VAS score.

### Biochemical assessments

Blood samples were obtained from all participants, then centrifuged for 10 minutes at 3000 rpm and stored at -80 °C. Fasting serum glucose, triglyceride (TG), total cholesterol (TC), and high density lipoprotein (HDL) were measured by commercial kits (Pars Azmoon, Tehran, Iran). Serum low density lipoprotein (LDL) was calculated using Friedewald method. ^27^ Serum insulin level was measured using enzyme-linked immunosorbent assay (ELISA) kit (Bioassay Technology Laboratory, Shanghai Korean Biotech, Shanghai City, China). Homeostasis model assessment for insulin resistance index (HOMA-IR) and quantitative insulin sensitivity check index (QUICKI) were calculated according to the formula provided in a study by Tohidi.^28^ Plasma Ag-RP and α-MSH levels were assessed using commercially available ELISA kits (Bioassay Technology Laboratory, Shanghai Korean Biotech, Shanghai City, China) according to their protocols. The minimum detectable level of plasma Ag-RP and α-MSH were 1.03 pg/mL and 5.07 ng/L, respectively. Sensitivity, intra-assay, and inter-assay precision for Ag-RP test were 27 pg/mL, < 10%, and < 12%, respectively. Accordingly, sensitivity, intra-assay, and inter-assay precision for α-MSH test were 0.039 ng/L, < 8%, and < 10%, respectively.

### Dietary assessments

The validated semi-quantitative food frequency questionnaire (FFQ) with 132 food items was used to estimate dietary intakes.^29^ The amount of consumed food items and daily nutrient and energy intake were calculated according to Iranian food consumption table (FCT).^30^ Then, DQI-I was calculated based on the obtained information. The final scores of the index were classified as diet variety (score of 0-20), diet adequacy (score of 0-40), diet moderation (score of 0-30), and dietary balance (score of 0-10). The variety item included the degree of diversity in consumed food groups (e.g., meats/eggs, dairy products, legumes, cereals, fruits, and vegetables) and protein sources (red meat, chicken, fish, dairy, legumes, and eggs). The final score of adequacy item was obtained from adequate intake of fruits, vegetables, grain, fibers, iron, vitamin C, and calcium compared with recommended dietary allowance (RDA) using food pyramid. In the moderation item, specific food compounds (e.g., total fat, saturated fat, cholesterol, and sodium amounts of foods), that should be restricted in the diet, received negative scores. Dietary balance considered the ratio of macronutrients to fatty acid amounts. The final scores ranged from 0 to 100 for each category. A diet with higher DQI-I score denoted a high-quality diet. ^31^

### Statistical analysis

Data were analyzed using SPSS software (SPSS Inc., Chicago, IL, USA, version 25.0). Normal distribution of variables was checked according to mean, standard deviation (SD), skewness, and kurtosis. Mean ± SD and median (min, max) of variables were used to report normal and non-normal data, respectively. Categorical values were reported as number (percent %) using χ2 test. Comparison of quantitative and metabolic biomarkers in different BMI and DQI-I tertiles was performed using one-way analysis of variance (ANOVA) and Kruskal-Wallis tests, respectively. Moreover, partial correlation test was used to identify the correlation of Ag-RP and α-MSH with cardiometabolic parameters in different DQI-I tertiles after adjusting for confounding factors, including age and BMI. *P* values less than 0.05 were considered as significant.

## Results

**Figure 1:**
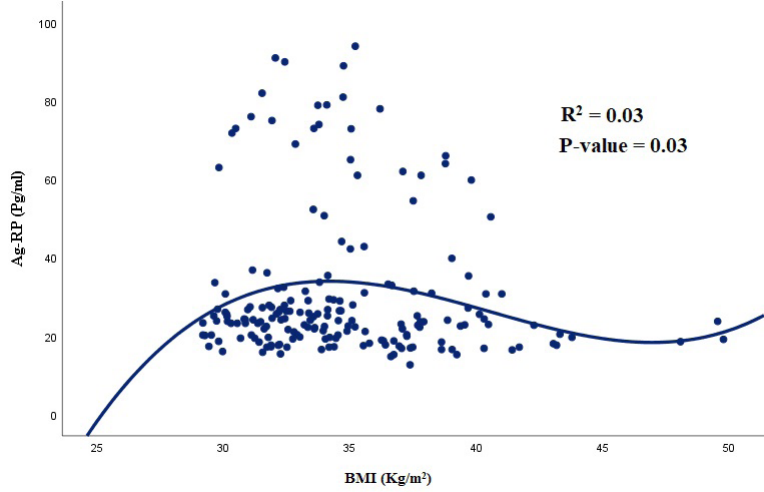
The correlation between Ag-RP and BMI levels

**Figure 2:**
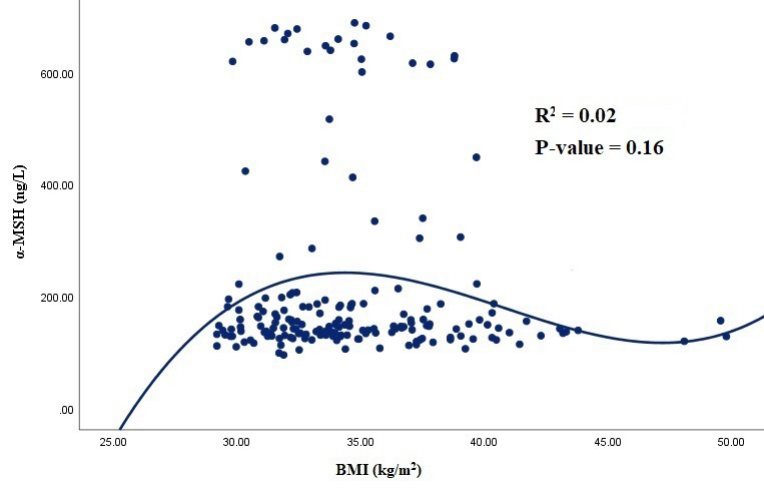
The correlation between α-MSH and BMI levels

In this study, out of 188 participants, 95 (51.1%) were male. The demographic and cardiometabolic parameters among different BMI tertiles are presented in Table 1. Accordingly, subjects in the highest tertile of BMI had a lower physical activity level. Also, people with low BMI had moderate to good SES. All the anthropometric parameters, appetite, SBP, and DBP were significantly different among BMI tertiles (*P* < 0.05), whereas SES and physical activity levels were not significantly different (*P* > 0.05). Among biochemical variables, plasma Ag-RP showed a significant difference among different BMI tertiles (*P* = 0.02), and those with a moderate BMI had higher levels of Ag-RP. In addition, subjects with a high level of BMI had elevated levels of TG, TC, LDL, and glucose, although the difference was not significant. Moreover, our findings indicated a non-significant difference between males and females in terms of DQI-I score (e.g., 4.0 ± 1.6 and 4.0 ± 1.3 for females and males, respectively). The results of the comparison of metabolic factors in different DQI-I tertiles are presented in Table 2. According to the results, subjects with high adherence to DQI-I had better metabolic panel of serum lipids and glycemic markers. Similarly, Ag-RP and α-MSH showed no significant difference among different tertiles of DQI-I in both males and females (*P* > 0.05). According to the results of the correlation analysis, Ag-RP and α-MSH were significantly correlated with metabolic parameters among groups with different adherence to DQI-I (Tables 3 and 4). In those with poor adherence to DQI-I, Ag-RP revealed a positive correlation with SBP (*P* = 0.03) among females even after adjusting for potential confounders, including age and BMI. Nonetheless, Ag-RP was positively correlated with WC (*P* = 0.01), SBP (*P* = 0.01), and DBP (*P* = 0.009) among males with moderate adherence to DQI-I. Similarly, α-MSH showed a positive correlation with insulin (*P* = 0.04) and a negative correlation with LDL (*P* = 0.03) among males and females with moderate adherence to DQI-I, respectively. In addition, α-MSH showed a significant positive correlation with weight (*P* = 0.03), WC (*P* = 0.007), SBP (*P* = 0.02), and DBP (*P* = 0.01) among males after adjusting for confounders in moderate adherence to DQI-I. On the other hand, in those with high adherence to DQI-I, no significant correlation was found between Ag-RP, α-MSH, and biochemical variables in both genders. Figure 1 shows a significant and nonlinear correlation between Ag-RP (R^2^ = 0.03; *P* = 0.03) and BMI levels among all participants, but α-MSH (R^2^ = 0.02; *P* = 0.16) failed to show any significant correlation with BMI levels (Figure 2).

**Table 1:** The comparison of baseline characteristics and biochemical parameters among BMI tertiles

	**BMI tertiles**			
	**BMI<32.42** **(kg/m** ^2^ **)** **(Mean±SD)**	**BMI (32.43-35.56)** **(kg/m** ^2^ **)** **(Mean±SD)**	**BMI>35.57** **(kg/m** ^2^ **)** **(Mean±SD)**	***P*** **value** ^a^
Age (years)	36.72 ± 6.98	36.69 ± 7.00	40.69 ± 7.80	0.002^*^
Gender, (%)				0.002^*^
Males	36.5	41.7	21.9	
Females	29.3	25.0	45.7	
Physical activity (min/week), (%)				0.28
Low	30.0	33.3	36.7	
Moderate	42.3	25.0	32.7	
High	28.3	43.5	28.3	
Socioeconomic status (SES), (%)				0.01^*^
Low	0.0	20.0	80.0	
Moderate	31.3	28.3	40.4	
High	36.1	41.0	22.9	
Marital status, (%)				0.24
Single	38.5	42.3	19.2	
Married	32.1	32.1	35.8	
Appetite	31.41 ± 8.84	36.09 ± 9.34	33.20 ± 8.09	0.01^*^
Weight (kg)	88.06 ± 10.15	96.69 ± 9.52	103.24 ± 13.76	<0.001^*^
Height (cm)	168.25 ± 9.50	168.71 ± 8.73	162.21 ± 9.22	<0.001^*^
FM (kg)	27.45 ± 5.42	31.30 ± 5.49	42.57 ± 8.22	<0.001^*^
FFM (kg)	60.62 ± 12.09	65.43 ± 12.68	60.68 ± 11.83	0.04^*^
FAT %	31.67 ± 7.60	32.30 ± 8.42	41.45 ± 6.52	<0.001^*^
WC (cm)	102.30 ± 8.63	109.31 ± 6.38	114.70 ± 10.30	<0.001^*^
HC (cm)	110.43 ± 4.55	114.61 ± 4.38	124.85 ± 7.47	<0.001^*^
WHR	0.92 ± 0.07	0.95 ± 0.06	0.91 ± 0.07	0.02^*^
SBP (mm Hg)	109.16 ± 17.41	115.03 ± 12.26	122.38 ± 16.65	<0.001^*^
DBP (mm Hg)	72.83 ± 11.93	75.23 ± 10.62	80.76 ± 13.01	0.001^*^
TC (mg/dL)	185.57 ± 35.10	188.38 ± 32.18	191.23 ± 34.16	0.64
HDL (mg/dL)	45.40 ± 9.17	45.12 ± 8.82	44.38 ± 8.67	0.80
LDL (mg/dL)	117.75 ± 32.11	118.82 ± 29.54	121.75 ± 31.41	0.75
TG (mg/dL)	98.00 (79.00, 132.00)	111.00 (80.00, 153.00)	111.00 (87.00, 144.00)	0.41
Glucose (mg/dL)	90.00 (85.00, 96.5)	90.00 (84.00, 99.00)	92.00 (85.00, 104.00)	0.20
Insulin (U/mL)	11.40 (9.05, 18.45)	13.20 (8.90, 23.30)	16.40 (9.20, 25.20)	0.51
HOMA-IR	2.62 (1.92, 4.14)	3.10 (1.88, 5.03)	3.72 (1.95, 6.27)	0.34
QUICKI	0.32 ± 0.02	0.32 ± 0.02	0.32 ± 0.03	0.42
Ag-RP (Pg/ml)	1.42 ± 0.18	1.48 ± 0.22	1.39 ± 0.18	0.02^*^
Alpha-MSH (ng/l)	2.24 ± 0.21	2.29 ± 0.26	2.23 ± 0.21	0.24

Abreviations: BMI, body mass index; FM, fat mass; FFM, fat free mass; WC, waist circumference; HC, hip circumference; WHR, waist to hip ratio; BMR, basal metabolic rate; SBP, systolic blood pressure; DBP, diastolic blood pressure, TG, triglyceride; TC, total cholesterol, HDL, high density lipoprotein; LDL, low density lipoprotein; HOMA-IR, homeostasis model assessment insulin resistance index; QUICKI, quantitative insulin sensitivity check index; Ag-RP, agouti related peptide; α-MSH, α-Melanocyte stimulating hormone. Normally distributed values are presented based on mead ± SD and abnormal values were reported as median (25 and 75 percentile). ^a^ *P*values are based on One-Way ANOVA. *P < 0.05

**Table 2:** Comparison of cardiometabolic factors in obese individuals in DQI-I tertiles

	**DQI-I tertile (Men)**		**DQI-I tertile (Women)**	
	**T1**	**T2**	**T3**	***P*** **value** ^a^	**T1**	**T2**	**T3**	***P *** **value** ^a^
TC (mg/dL)	192.21 ± 32.05	188.77 ± 29.98	187.71 ± 32.13	0.711	185.97 ± 31.5	183.37 ± 41.68	194.32 ± 35.48	0.461
HDL (mg/dL)	42.61 ± 6.42	43.17 ± 9.33	41.88 ± 7.55	0.920	50.77 ± 8.03	45.48 ± 9.57	45.97 ± 9.72	0.053
LDL (mg/dL)	121.43 ± 27.38	121.57 ± 28.04	118.14 ± 27.09	0.666	114.74 ± 29.84	118.45 ± 38.27	125.26 ± 33.41	0.453
TG (mg/dL)	121.0 (90.5, 166.50)	116.0 (73.0, 159.0)	114.5 (88.5, 191.75)	0.282	102.3 ± 36.11	97.19 ± 39.99	115.47 ± 45.18	0.195
Glucose (mg/dL)	98.30 ± 20.82	95.06 ± 15.33	93.25 ± 13.42	0.707	93.37 ± 11.99	89.04 ± 12.18	91.94 ± 9.09	0.329
Insulin (U/mL)	13.3 (9.6, 20.8)	12.0 (9.0, 23.1)	10.35 (7.88, 19.15)	0.442	14.85 ± 10.40, 19.15)	20.7 (11.70, 27.10)	12.25 (7.95, 24.53)	0.186
HOMA-IR	3.2 (2.06, 4.86)	2.84 (1.94, 5.12)	2.38 (1.62, 4.22)	0.423	3.57 (2.31, 4.79)	4.55 (2.83, 6.34)	2.76 (1.7, 5.6)	0.283
QUICKI	0.32 ± 0.03	0.33 ± 0.03	0.34 ± 0.03	0.317	0.33 ± 0.03	0.32 ± 0.03	0.33 ± 0.03	0334
α-MSH (ng/l)	149.0 (131.25, 233.45)	167.9 (127.0, 210.3)	160.15 (135.25, 194.93)	0.809	140.5 (126.0, 208.53)	140.0 (128.5, 162.9)	140.75 (127.5, 155.9)	0.842
Ag-RP (Pg/ml)	24.3 (21.80, 47.40)	26.40 (22.80, 33.60)	26.70 (21.75, 31.40)	0.756	22.15 (17.5, 33.33)	22.2 (20.20, 25.10)	22.45 (17.58, 25.13)	0.902

Normally distributed values are presented based on mead ± SD and abnormal values were reported as median (min, max). ^a^ *P* values based on One-Way ANOVA and Kruskal-Wallis test for normal and abnormal variables, respectively. *P < 0.05

**Table 3:** The correlation between Ag-RP with biochemical variables in DQI-I tertiles in a sex-stratified model

		**Ag-RP (Males)**		**Ag-RP (Females)**	
**Study groups**	**Variables**	**r**	***P *** **value** ^a^	**r**	***P *** **value** ^a^
DQI-I T1	Weight (kg)	0.19	0.29	0.20	0.29
	FM	-0.21	0.25	0.17	0.38
	FFM	0.30	0.09	0.08	0.66
	FAT%	-0.28	0.11	0.07	0.72
	WC	-0.16	0.37	0.26	0.17
	WHR	-0.12	0.50	0.04	0.82
	SBP (mm Hg)	-0.23	0.20	0.40	0.03^*^
	DBP (mm Hg)	-0.25	0.15	0.06	0.75
	TG (mg/dL)	-0.13	0.47	0.14	0.47
	TC (mg/dL)	-0.26	0.15	0.09	0.64
	HDL (mg/dL)	-0.17	0.34	-0.36	0.05
	LDL (mg/dL)	-0.18	0.31	0.17	0.38
	Glucose (mg/dL)	-0.13	0.48	-0.04	0.83
	Insulin (U/mL)	0.25	0.16	-0.19	0.31
	HOMA-IR	0.04	0.82	-0.19	0.32
	QUICKI	-0.18	0.33	0.27	0.15
DQI-I T2	Weight (kg)	0.29	0.09	-0.09	0.66
	FM	0.24	0.17	0.04	0.83
	FFM	0.17	0.32	-0.20	0.32
	FAT%	0.09	0.59	0.10	0.62
	WC	0.43	0.01^*^	0.04	0.84
	WHR	0.14	0.42	0.17	0.39
	SBP (mm Hg)	0.42	0.01^*^	0.03	0.87
	DBP (mm Hg)	0.44	0.009^*^	0.23	0.26
	TG (mg/dL)	0.16	0.35	-0.14	0.49
	TC (mg/dL)	0.10	0.58	-0.13	0.51
	HDL (mg/dL)	0.03	0.86	0.10	0.62
	LDL (mg/dL)	0.02	0.88	-0.14	0.49
	Glucose (mg/dL)	-0.14	0.42	-0.13	0.51
	Insulin (U/mL)	-0.20	0.26	0.09	0.65
	HOMA-IR	-0.22	0.20	0.03	0.86
	QUICKI	0.21	0.23	-0.09	0.63
DQI-I T3	Weight (kg)	-0.001	0.99	0.003	0.98
	FM	0.12	0.56	0.08	0.64
	FFM	-0.10	0.64	-0.07	0.67
	FAT%	0.15	0.48	0.21	0.23
	WC	0.01	0.93	-0.09	0.60
	WHR	0.08	0.71	-0.06	0.71
	SBP (mm Hg)	0.20	0.36	-0.22	0.22
	DBP (mm Hg)	0.19	0.39	-0.13	0.48
	TG (mg/dL)	0.006	0.97	-0.20	0.26
	TC (mg/dL)	0.07	0.73	-0.12	0.50
	HDL (mg/dL)	0.13	0.55	-0.08	0.63
	LDL (mg/dL)	0.04	0.83	-0.04	0.80
	Glucose (mg/dL)	0.13	0.54	-0.10	0.56
	Insulin (U/mL)	-0.24	0.28	-0.08	0.64
	HOMA-IR	-0.23	0.29	-0.04	0.81
	QUICKI	0.14	0.50	-0.008	0.96

Abbreviations: Ag-RP, agouti related peptide; α-MSH, α-melanocyte stimulating hormone; SBP, systolic blood pressure; DBP, diastolic blood pressure, TG, triglyceride; TC, total cholesterol, HDL, high density lipoprotein; LDL, low density lipoprotein; HOMA-IR, homeostasis model assessment insulin resistance index; QUICKI, quantitative insulin sensitivity check index. ^a^ *P*values are based on partial correlation. *P < 0.05

**Table 4:** The correlation between α-MSH with biochemical variables in DQI-I tertiles in a sex-stratified model

		**α-MSH (Males)**		**α -MSH (Females)**	
**Study groups**	**Variables**	**r**	***P *** **value** ^a^	**r**	***P *** **value** ^a^
DQI-I T1	Weight (kg)	0.02	0.89	0.17	0.37
	FM	-0.24	0.17	0.16	0.40
	FFM	.15	0.40	0.04	0.83
	FAT%	-0.24	0.17	0.10	0.60
	WC	-0.22	0.23	0.24	0.21
	WHR	-0.10	0.59	0.03	0.87
	SBP (mm Hg)	-0.24	0.18	0.35	0.06
	DBP (mm Hg)	-0.25	0.16	0.02	0.90
	TG (mg/dL)	-0.05	0.75	-0.13	0.49
	TC (mg/dL)	-0.20	0.28	0.06	0.76
	HDL (mg/dL)	0.02	0.88	0.16	0.39
	LDL (mg/dL)	-0.21	0.23	-0.40	0.03
	Glucose (mg/dL)	-0.19	0.28	0.14	0.47
	Insulin (U/mL)	0.36	0.04^*^	-0.23	0.22
	HOMA-IR	0.15	0.39	-0.24	0.20
	QUICKI	-0.31	0.08	0.32	0.09
DQI-I T2	Weight (kg)	0.37	0.03^*^	0.01	0.96
	FM	0.25	0.14	0.14	0.47
	FFM	0.25	0.15	-0.11	0.57
	FAT%	0.08	0.62	0.08	0.68
	WC	0.46	0.007^*^	0.15	0.46
	WHR	0.19	0.27	0.21	0.29
	SBP (mm Hg)	0.40	0.02^*^	0.13	0.50
	DBP (mm Hg)	0.42	0.01^*^	0.28	0.16
	TG (mg/dL)	-0.10	0.57	-0.10	0.62
	TC (mg/dL)	0.05	0.76	-0.19	0.35
	HDL (mg/dL)	0.07	0.66	-0.06	0.74
	LDL (mg/dL)	0.11	0.54	0.12	0.56
	Glucose (mg/dL)	-0.01	0.95	-0.22	0.27
	Insulin (U/mL)	-0.03	0.83	0.13	0.51
	HOMA-IR	-0.06	0.72	0.06	0.75
	QUICKI	0.06	0.71	0.16	0.43
DQI-I T3	Weight (kg)	-0.10	0.63	-0.18	0.32
	FM	0.03	0.88	-0.005	0.97
	FFM	-0.17	0.43	-0.29	0.10
	FAT%	0.14	0.51	0.32	0.07
	WC	-0.14	0.53	-0.10	0.57
	WHR	-0.12	0.56	-0.06	0.72
	SBP (mm Hg)	0.34	0.11	-0.25	0.16
	DBP (mm Hg)	0.27	0.21	-0.06	0.72
	TG (mg/dL)	0.15	0.48	-0.20	0.26
	TC (mg/dL)	0.10	0.62	0.11	0.54
	HDL (mg/dL)	0.01	0.93	-0.30	0.08
	LDL (mg/dL)	0.19	0.37	0.05	0.75
	Glucose (mg/dL)	0.06	0.77	0.18	0.31
	Insulin (U/mL)	-0.34	0.11	-0.04	0.79
	HOMA-IR	-0.36	0.09	-0.03	0.85
	QUICKI	0.28	0.19	0.05	0.77

Ag-RP, agouti related peptide; α-MSH, α-melanocyte stimulating hormone; SBP, systolic blood pressure; DBP, diastolic blood pressure, TG, triglyceride; TC, total cholesterol, HDL, high density lipoprotein; LDL, low density lipoprotein; HOMA-IR, homeostasis model assessment insulin resistance index; QUICKI, quantitative insulin sensitivity check index ^a^ *P* values are based on partial correlation *P < 0.05

## Discussion

The present cross-sectional study revealed the relationship between Ag-RP, α-MSH, and metabolic parameters in obese individuals with different adherence to dietary quality index. We observed significant differences in anthropometric parameters in different BMI tertiles. In addition, appetite, SBP, and DBP were significantly increased in higher levels of BMI. Similarly, Sushith S et al reported a higher level of BMI in hypertensive versus non-hypertensive patients with type 2 diabetes.^32^ Consistent with our results, Luo et al revealed that Ag-RP neuropeptide might be a potent mediator between appetite and obesity state.^5^ Also, similar to the study by Masoodian et al, there was no significant difference in glycemic markers and lipid profile between different BMI tertiles^33^ Based on our findings, Ag-RP and α-MSH levels were higher among higher tertiles of BMI; other evidence has also indicated that obese adults have elevated plasma levels of α-MSH and Ag-RP.^34,35^ It seems that obesity may induce hyperleptinemia by inducing resistance against α-MSH and Ag-RP.^36,37^ Ag-RP may affect leptin and cause hyperphagia in obesity.^19^ As the main findings of this study, we should highlight the correlation of Ag-RP and α-MSH with cardiometabolic parameters. It is also suggested that fatty acids affect Ag-RP expression through activation of peroxisome proliferator activated receptor (PPARγ) and sterol regulatory element-binding proteins (SREBP).^37^ According to our findings, a positive correlation was found between α-MSH and insulin level in male participants with poor adherence to DQI-I. Similarly, Katuski et al reported that plasma concentration of α-MSH significantly correlated with fasting insulin levels in males.^38^ It has been shown that α-MSH as a potent inducer of hyperglycemia in mice. In another study by Yaswen et al, α-MSH inhibited cellular uptake of free fatty acids and stimulated the lipolysis among α-MSH-knockout mice. In our study, a positive correlation was reported between α-MSH and WC by increasing adherence to DQI-I in males. Previously, it was shown that α-MSH was correlated with visceral fat area in obese male subjects.^15^ Recent studies demonstrated that there is an increased intraperitoneal adipose tissue expression of several cytokine complements and α-MSH. In contrast, Donahoo et al did not report such an association among males.^39^ Moreover, in *ex-vivo* experiments, obesity caused endoplasmic reticulum stress, reduced pro-converting enzyme 2 production, and catalyzed the conversion of adrenocorticotropin to α-MSH. Consequently, it contributes to energy balance regulation in obesity.^8^

We also observed a straight linear association between Ag-RP and BMI in those with BMI less than 35 kg/m^2^; however, in people with a BMI more than 35 kg/m^2^, the linear trend reduced and the figure reached to a plateau status. This can be attributed to the physiological feedback of human body to maintain body weight. In contrast, α-MSH failed to show a significant correlation with BMI level. In a study by Donaho et al, plasma concentrations of α-MSH were significantly and positively correlated with BMI only among obese individuals.^39^ Although the exact underlying mechanisms of these associations are not fully understood, prior studies have indicated that Ag-RP increases the ability of IL-1β to enhance adrenocorticotropic hormone (ACTH), suggesting the possible pro-inflammatory role of Ag-RP and development of obesity and type 2 diabetes.^37,40,41^ The current study had some limitations. First, due to the cross-sectional design of the study, we failed to investigate the causal relationships between different variables. Second, dietary assessment among obese individuals is a matter of bias due to their possible under-reporting. Third, the present study was conducted in northwestern region of Iran with special dietary patterns and eating habits. Hence, the results cannot be generalized to other populations, even though we used validated questionnaires to reduce the risk of bias.

## Conclusion

Our findings indicated that SBP, DBP, and WC were correlated with Ag-RP among obese individuals with poor and moderate adherence to DQI-I in a sex-stratified model. Also, Ag-RP had a positive association with BMI. Further studies with experimental and longitudinal designs are suggested to better explore the role of these neuropeptides in pathogenesis of obesity and obesity-related comorbidities.

## Acknowledgements

The authors wish to thank all the study participants.

## Competing interest

The authors declare that there is no conflict of interest.

## Funding

The current study was granted by Tabriz University of Medical Sciences (Grant number: 64111; code: IR.TBZMED.REC.1399.062 and grant number: 67115; code: IR.TBZMED.REC.1400.454).

## Ethical approval

The study protocol was approved by the Ethics Committee of Tabriz University of Medical Science (Ethics number: IR.TBZMED.REC.1399.062 and IR.TBZMED.REC.1400.454).
